# Knowing Christ

**Published:** 1890-01-11

**Authors:** 


### Words of Consolation.

### KNOWING CHRIST.

*(For Reading to the Sick.)*

"Who lives at the great house on the hill yonder?" asked a stranger, of a passing labourer, as he walked through the little village of N——. "The Squire," was the reply. "And what sort of man is he?" continued the gentleman. "Oh, I don't know," said the man; "I never saw him in my life. He never comes near us except to take his rents, and I never go near him. Some folks say he is mortal kind, and would do anything for you if you only ask him, but a good many would tell you quite a different tale; that he is a hard master, and will have his dues. His steward lets us know fast enough when he is coming for his rent, and then you must pay up, or it will be the worse for you." "But," said the stranger, again, "have you ever been behind with your rent? and did you not go and tell him how it was illness or trouble which kept you from paying, surely he was not hard on you then?" The man shook his head, and gave a cunning grin. "Aye, I did once make up a good story for the steward, but the Squire dropped upon me pretty sharp. He said I was lazy, and drank; did not send my children to school, and let my mother go to the workhouse." "Was it the truth?" asked the gentleman. "Well, a poor man must have his drop of beer; and if his missus don't keep the brats at school, he can't help it, and times are too bad to let you keep your mother too. But there—I don't care. I don't know the Squire, and don't want to. He can't get blood out of a stone, and so he'll have to give in—it'll all be right in the end."

The man seemed a lazy, worthless sort of fellow, who only cared for himself. So the stranger said "Good evening," and passed on, the man's words weighing on his mind. How many, he thought, with a sigh, are like this clown in their behaviour towards Christ! They do not know Him, and so, in their ignorance, call Him a hard master, because He would have them use the talents He has given them for His honour. They wrap up their one talent in a napkin, instead of making it into ten, and sit down contentedly, saying, "It will be all right in the end." His ministers say He is merciful and kind. He will not punish us if we have done no harm. We are not clever, nor rich, nor handsome, nor good-tempered as some people we know, and so if we do beat our fellow-servants, and eat and drink, and are drunken, it is not our own fault.

But, alas, my friends, what a frightful mistake you are making! God expects you to do the most with your opportunities, be they little or great, and if you cannot do it of yourselves He wishes you to ask in Christ's name and He will help you. To ask Him you must first know Him, and then go into His presence and fall low on your knees before His footstool. Do you personally, really know Him? Do you know Him as your Saviour? Have you ever looked on Him whom they pierced, and seen the nail-prints in His blessed hands and feet which 1300 "years ago were nailed for our advantage on the bitter cross." Have you gazed at the crown of thorns which He wears as a monarch and a conqueror, and, gazing, have you prayed, "Lord, I believe, help Thou mine unbelief; Lord, I believe that Thou art the Christ, the Son of the living God; in the hour of death, and in the day of judgment, Good Lord deliver us!" It is the want of knowing Him which will be our perdition. Try to know Him in His Word and Sacraments; try to make your life like His. See and know Him in His suffering children; see and know Him in yourselves. In your sorrows and your tears you resemble Him, for He bare our griefs and carried our sorrows. Keep your eyes fixed firmly on Him till you pierce the veil; then, having known Him here on earth, you will recognise Him when He comes in glory.

Those dear tokens of His passion,
Still His dazzling body bears,
Cause of endless exultation
To His ransomed worshippers.

He will come sooner or later to us all, and at the last day "every eye shall see Him, and they a lso which pierced Him."

